# Dihydromyricetin supplementation improves ethanol-induced lipid accumulation and inflammation

**DOI:** 10.3389/fnut.2023.1201007

**Published:** 2023-08-23

**Authors:** Isis Janilkarn-Urena, Alina Idrissova, Mindy Zhang, Masha VanDreal, Neysa Sanghavi, Samantha G. Skinner, Sydney Cheng, Zeyu Zhang, Junji Watanabe, Liana Asatryan, Enrique Cadenas, Daryl L. Davies

**Affiliations:** ^1^Titus Family Department of Clinical Pharmacy, University of Southern California Alfred E. Mann School of Pharmacy and Pharmaceutical Sciences, Los Angeles, CA, United States; ^2^Translational Research Lab, USC Alfred E. Mann School of Pharmacy and Pharmaceutical Sciences, University of Southern California, Los Angeles, CA, United States; ^3^Department of Neurosurgery, Cedars-Sinai Medical Center, Los Angeles, CA, United States

**Keywords:** Dihydromyricetin, polyphenols, flavonoids, ethanol, lipids, inflammation, lipophagy

## Abstract

**Introduction:**

Excessive alcohol consumption leads to a myriad of detrimental health effects, including alcohol-associated liver disease (ALD). Unfortunately, no available treatments exist to combat the progression of ALD beyond corticosteroid administration and/or liver transplants. Dihydromyricetin (DHM) is a bioactive polyphenol and flavonoid that has traditionally been used in Chinese herbal medicine for its robust antioxidant and anti-inflammatory properties. It is derived from many plants, including *Hovenia dulcis* and is found as the active ingredient in a variety of popular hangover remedies. Investigations utilizing DHM have demonstrated its ability to alleviate ethanol-induced disruptions in mitochondrial and lipid metabolism, while demonstrating hepatoprotective activity.

**Methods:**

Female c57BL/6J mice (*n* = 12/group) were treated using the Lieber DeCarli forced-drinking and ethanol (EtOH) containing liquid diet, for 5 weeks. Mice were randomly divided into three groups: (1) No-EtOH, (2) EtOH [5% (v/v)], and (3) EtOH [5% (v/v)] + DHM (6 mg/mL). Mice were exposed to ethanol for 2 weeks to ensure the development of ALD pathology prior to receiving dihydromyricetin supplementation. Statistical analysis included one-way ANOVA along with Bonferroni multiple comparison tests, where *p* ≤ 0.05 was considered statistically significant.

**Results:**

Dihydromyricetin administration significantly improved aminotransferase levels (AST/ALT) and reduced levels of circulating lipids including LDL/VLDL, total cholesterol (free cholesterol), and triglycerides. DHM demonstrated enhanced lipid clearance by way of increased lipophagy activity, shown as the increased interaction and colocalization of p62/SQSTM-1, LC3B, and PLIN-1 proteins. DHM-fed mice had increased hepatocyte-to-hepatocyte lipid droplet (LD) heterogeneity, suggesting increased neutralization and sequestration of free lipids into LDs. DHM administration significantly reduced prominent pro-inflammatory cytokines commonly associated with ALD pathology such as TNF-α, IL-6, and IL-17.

**Discussion:**

Dihydromyricetin is commercially available as a dietary supplement. The results of this proof-of-concept study demonstrate its potential utility and functionality as a cost-effective and safe candidate to combat inflammation and the progression of ALD pathology.

## 1. Introduction

Alcohol use disorder (AUD) affects over 280 million people worldwide, and in the United States alone, it affects over 18 million people, leading to approximately 140,000 deaths annually (1, 2). This ranks AUD third on the list of preventable causes of death and morbidity. Unfortunately, the rates of alcohol misuse are on the rise, with unhealthy drinking patterns contributing to a higher incidence of mortality, particularly due to alcohol-associated liver disease (ALD) (3–5). The liver is the primary site of alcohol metabolism, and when ALD manifests, it is in a progressive order. This progression includes alcohol-associated fatty-liver disease (AFLD), alcohol-associated steatohepatitis (ASH) and fibrosis, to ultimately cirrhosis. The stages of ALD are characterized by disruptions in lipid metabolism and transport, altering the levels of free fatty acids, triglycerides, total cholesterol, and lipoproteins that result in injury due to lipotoxicity, oxidative stress, and inflammation (6). Available FDA-approved medications have limited success in treating patients for AUD, and there are no approved pharmaceutical or nutritional therapies for ameliorating ALD beyond the administration of corticosteroids as anti-inflammatory agents or in the worst-case scenarios, a liver transplant (7). The lack of effective therapies for ALD is due, in part, to the multifactorial systemic responses that are associated with heavy ethanol (EtOH) intake and the multi-organ damage that can result from excessive EtOH consumption.

Plant-derived products, including members of the polyphenol families, are traditionally used worldwide for the treatment of liver disorders that include hepatic-driven metabolic imbalances (8–10). Polyphenols can regulate homeostasis by acting on nuclear receptors in response to the cellular environment and metabolic sensors. Emerging studies have demonstrated the effects of dietary polyphenols on dyslipidemia by reducing circulating levels of low-density lipoprotein (LDL), very low-density lipoprotein (VLDL), and promoting high density lipoprotein (HDL) levels, while improving liver function as noted by improved aspartate and alanine aminotransferase (AST and ALT) levels (10–13). Dihydromyricetin (DHM), a polyphenol and bioactive flavonoid found in many plants such as *Hovenia dulcis*, has been used for centuries in Traditional Chinese Medicine and is still used today (14), namely, as the active ingredient of popular hangover remedies.

In 2021, the global natural product (i.e., herbal medicine) market was valued at nearly $152 billion, highlighting the growing consumer preference for natural remedies over synthetic products (15, 16). In fact, the market for hangover cures was valued at $1.8 billion in 2021, and is expected to grow over 14.6% by 2028 (17), led by plant-based herbal products. Building evidence suggests that DHM improves steatosis (18–20) while providing hepatoprotective effects and restoring metabolic processes (19, 21). Regarding alcohol, commercially available DHM is used for its anti-veisalgia effect and is instructed to be administered before, during, and after consuming large amounts of ethanol.

To expand on this further, our group has begun to investigate the effects of DHM on ethanol-induced disturbances in lipid metabolism, steatosis, and inflammation. We recently reported that administration of 5 and 10 mg/kg of DHM delivered via intraperitoneal (i.p.) injection significantly protected the livers of mice from ethanol-induced steatosis and improved mitochondrial health via the AMP-activated protein kinase (AMPK), sirtuin-1 (SIRT1), PPARG coactivator-1α (PGC-1α) signaling pathway (18, 21). The AMPK-SIRT1-PGC-1α pathway is a key regulator of energy homeostasis through its effects on metabolic and mitochondrial activity, namely, lipid oxidation, mitochondrial biogenesis, and autophagy (22–26). Autophagy is an evolutionarily conserved process that plays an important role in liver physiology, and is induced through AMPK pathway activation (27). Typically, autophagy promotes the proteolytic degradation and recycling of damaged proteins and organelles, including lipid droplets (LDs), in response to environmental cues, such as starvation and energy requirements. LD catabolism is mediated by lipolysis and lipophagy, a form of selective macro-autophagy that targets lipid droplets (28).

Along with steatosis, inflammation plays a critical role in the development and progression of ALD. Chronic alcohol consumption leads to the activation of several inflammatory pathways including NF-κB and toll-like receptor 4 (TLR4) signaling pathways, and inflammasome activation (29). These pathways are responsible for elevation of pro-inflammatory cytokines which promote liver inflammation and injury via increased oxidative stress and mitochondrial dysfunction (30). Furthermore, chronic alcohol consumption leads to disruption of the gut barrier, leading to bacterial translocation and release of endotoxins into the liver. These endotoxins activate Kupffer cells, which leads to further increases in production of pro-inflammatory cytokines and oxidative stress resulting in exacerbated liver inflammation and injury (31). Overall, the inflammatory response in ALD is a complex process which involves multiple cell types, mediators, and pathways. Targeting inflammatory responses early could prove to be an important therapeutic strategy for ALD.

Alcohol-associated fatty-liver disease and ASH are characterized by the accumulation of fat primarily found in the form of lipid droplets and increased inflammatory signaling through TNF-α, IL-1β, IFN-γ, IL-17, and IL-6 (32, 33). In the current investigation, we tested the hypothesis that oral DHM improves ethanol-induced disruptions in lipid homeostasis by reducing levels of harmful lipids, leading to decreased levels of circulating pro-inflammatory cytokines.

## 2. Materials and methods

### 2.1. Lieber DeCarli Diet (LDC)

Female wild-type c57BL/6J mice (Jackson Laboratories, Bar Harbor, ME) weighing ≥ 19 g and ≥ 10 weeks of age at the beginning of the study were individually housed in cages with shredded filter paper and wooden blocks for enrichment and to prevent malocclusion from receiving a liquid-only diet. Mice were acclimated for 2 weeks in temperature (22°C), light, and humidity-controlled (40–60%) conditions with a 12 h light/dark cycle. During acclimation weeks, mice were given free access to the liquid Lieber DeCarli diet with no ethanol (Bio-Serv, Flemington, NJ, USA) following the model described by Bertola et al. (34), with modifications. After the acclimation period, mice were randomly assigned to groups (where feed was given *ad libitum*): (1) No-EtOH (*n* = 12); (2) EtOH [(*n* = 12) 5.5% (v/v)]-containing LDC diet; and (3) DHM (*n* = 12; 6 mg/mL) + EtOH-containing LDC diet, for a total of 5 weeks. Every morning, fluid intake was recorded by measuring the meniscus on the graduated feed tube. Mice in the DHM group were exposed to ethanol-only for 2 weeks prior to DHM supplementation, which lasted for the remainder of the study; the feeding paradigm was isocaloric between groups. DHM, [(2R, 3R)-3, 5, 7-trihydroxy-2-(3, 4, 5-trihydroxyphenyl)- 2,3-dihydrochromen-4-one], HPLC grade, >98%, MW 320.25 was purchased from Master Herbs Inc., Pomona, CA. The LDC diet is a robust forced-drinking model that induces severe liver disease with a potential for a high mortality rate. Therefore, for this proof-of-concept study, a single dose of 6 mg/mL was used as a comparison to the 10 mg/kg dose delivered via i.p. in our previous publications. After the study period ended, mice were euthanized via CO_2_ exposure followed by cardiac puncture. Blood was collected and kept at room temperature for 45 min and serum was separated by centrifugation for 10 min at 10,000 × *g* in 4°C and stored at −80°C until use; livers were harvested and frozen in nitrogen-isopentane and stored at −80°C until use or fixed in 10% formalin and embedded in paraffin. Animals used in the study were considered and handled in adherence to the University of Southern California’s Department of Animal Resources Institutional Animal Care and Use Committee (IACUC) policies and guidelines.

### 2.2. Immunohistochemistry

Lipid droplets were stained using Oil Red O Staining Kit (Lifeline Cell Technology, San Diego, CA, USA) on frozen liver sections (10 μm thick). Liver sections were also stained with Hematoxylin and Eosin (H&E) staining kit (Abcam, Boston, MA, USA). Antibodies against p62/SQSTM-1 (1:400, Cell Signaling Technology, Danvers, MA, USA); LC3B (1:1,000, Cell Signaling Technology, Danvers, MA, USA); PLIN-1 (1:200, Cell Signaling Technology, Danvers, MA, USA); anti-CD68 (1:250, Cell Signaling Technology, Danvers, MA, USA); and Alexa Fluor 405, 488, and 647 secondary antibodies (1:250, Cell Signaling Technology, Danvers, MA, USA) were used for visualization. Images were acquired using Cytation 5 Cell Imaging Multi-Mode Reader (BioTek, Winooski, VT, USA) and Zeiss LSM880 w/Airyscan Confocal Laser Scanning Microscope (Carl Zeiss Microscopy, White Plains, NY, USA), and were analyzed using ImageJ software (ImageJ; Coloc2 Fiji software) and Zen (Black and Blue versions) imaging analysis software (Carl Zeiss Microscopy, White Plains, NY, USA). Lipid droplet density and size were analyzed using whole image analysis on the ImageJ Color Threshold software.

### 2.3. Immunoblotting

Protein expression (Atg7, PLIN-1, p62, CETP, and LCAT) was analyzed using protein extracts (60–125 mg for immunoprecipitation) from liver homogenates that were isolated using Dynabeads Magnetic Beads (Thermo-Fisher Scientific, MA, USA), and visualized via sodium dodecyl sulfate-polyacrylamide gel electrophoresis (SDS-PAGE), where bands were detected by chemiluminescent reaction. Signal density was quantified by densitometry using ImageJ software: Atg7, PLIN-1, p62/SQSTM-1, CETP, and LCAT were analyzed using immunoprecipitation, while LC3B and Beta-actin antibodies purchased from Cell Signaling Technology, CA were analyzed from Western blots (diluted 1:1,000, while 10 μg of antibody was used for IP).

### 2.4. Biochemical assays

The following assays were measured from serum: aspartate and alanine aminotransferase (AST and ALT) levels were measured using AST and ALT activity assays (Sigma Aldrich, St. Louis, MO, USA). Free cholesterol and cholesteryl esters were measured using the Cholesterol Fluorometric Assay Kit (Cayman Chemical, Ann Arbor, MI, USA). LDL/VLDL levels were measured using the Cholesterol Assay Kit (Abcam, Boston, MA). Circulating triglyceride levels were measured from liver homogenates (∼100 mg) and serum using Triglyceride Colorimetric Assay Kit (Cayman Chemical, Ann Arbor, MI, USA).

Mitochondrial oxidative phosphorylation system (OXPHOS) in complexes I, II, and IV were analyzed using Complex I Enzyme Activity Colorimetric Assay Kit, Complex II Enzyme Activity Microplate Assay Kit, and the Complex IV Rodent Enzyme Activity Microplate Assay Kit (Abcam, Boston, MA, USA) using isolated mitochondria that were purified from the liver tissues using the Mitochondria Isolation Kit for Tissue (Abcam, Boston, MA, USA).

Cytokine levels were measured using Proteome Profiler Array Mouse Cytokine Array Kit Panel A (R&D Systems, Minneapolis, MN, USA) and signal density was quantified by densitometry using ImageJ software.

### 2.5. Statistical analysis

Immunohistochemistry images were analyzed using *n* = 3–4 from each group and 4 different sections were analyzed per sample. Biochemical assays were conducted using 3–4 samples from each group. Data are presented as means ± standard deviation. Statistical analysis included one-way ANOVA along with Bonferroni multiple comparison tests using Prism 9.3 (GraphPad Software, Inc., San Diego, CA, USA), where *p* ≤ 0.05 was considered statistically significant.

## 3. Results

### 3.1. DHM administration ameliorates ethanol-induced changes in hepatic and circulating lipid content while improving aminotransferase levels

**FIGURE 1:**
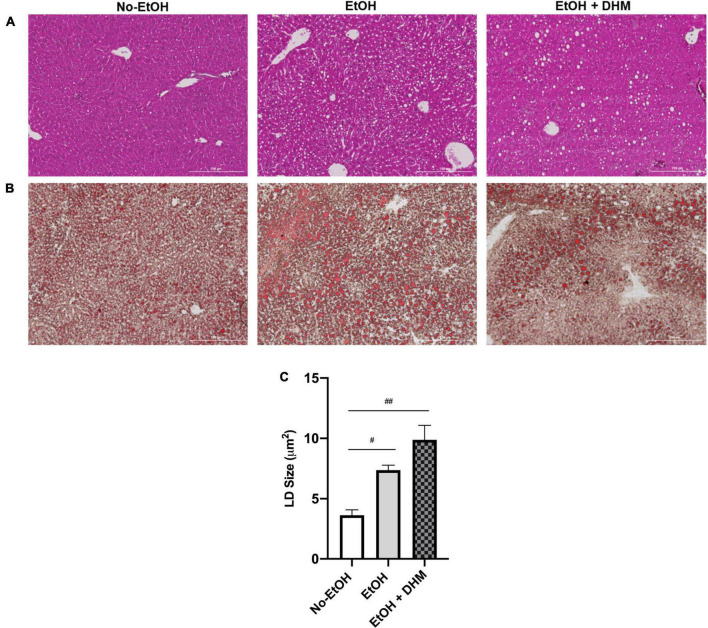
DHM administration results in heterogeneous lipid droplet size and distribution. Histology images (scale bars: 200 μm) shown are **(A)** Hematoxylin and Eosin and **(B)** Oil Red-O-stained liver sections demonstrating heterogeneity in LD size and distribution between groups (white circles); **(C)** Lipid droplet size in each group (^#^0.0063, ^##^< 0.0001).

To investigate the utility of oral DHM, mice in the DHM group received ethanol-only treatment for 2 weeks prior to DHM supplementation to assure the initiation and development of ALD pathology. A hallmark of early ALD is hepatic steatosis, characterized by the accumulation of LDs throughout the liver and disruptions in lipid homeostatic conditions (35–37). H&E and Oil Red O-staining of liver tissue sections demonstrated increased steatosis in the EtOH group which was alleviated by DHM treatment (Figures 1A, B; scale bars 200 μm). LDs are synthesized by nearly all cells, and size varies considerably among different cell types in response to environmental cues, particularly in the liver. Chronic ethanol consumption alters hepatocyte LD properties, including increased size and cellular distribution (38). Interestingly, mice in the DHM group exhibited a wide range of lipid accumulation and distribution in addition to significantly larger LDs, compared to all groups. In a previous study, it was shown that heterogeneous lipid distribution within the hepatocyte population, similar to what is observed in the DHM group, is a potential hepatoprotective social organization that reduces lipotoxicity within the overall region, compartmentalizing lipids within a cell population (39). The authors also reported that LD heterogeneity is not only reversible and variable (depending on intracellular-environmental factors) but also allows for the reduction of lipotoxicity between cells by exchanging LD content over time. We found a wide range of noticeable hepatocyte-to-hepatocyte LD heterogeneity, which was more prominent in the group receiving DHM. LD size was also found to be varied across groups, with the mean LD sizes in the No-EtOH group measuring at 3.64 μm^2^, EtOH-only at 7.37 μm^2^, and DHM measuring at 9.88 μm^2^. Mice receiving EtOH had larger LDs than the No-EtOH group (^#^0.0063; Figure 1C), and the difference was even greater in the mice fed DHM (^##^< 0.0001; Figure 1C).

**FIGURE 2:**
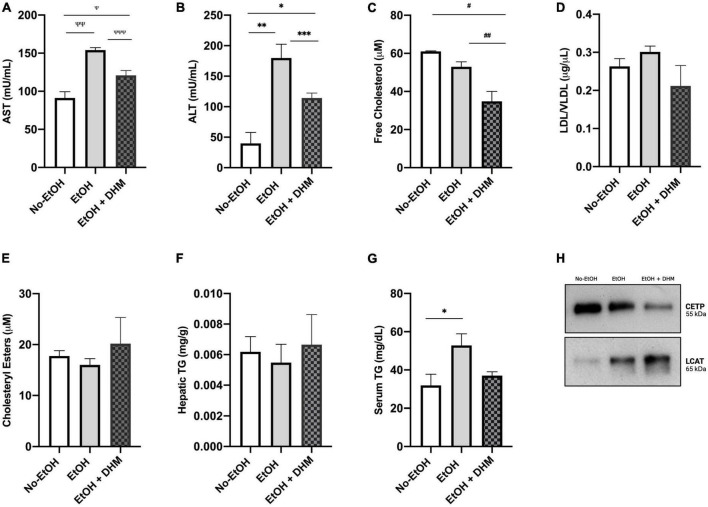
DHM administration ameliorates ethanol-induced changes in circulating and hepatic lipid content and improves aminotransferase levels. DHM effect on levels of circulating: **(A)** Aspartate aminotransferase (AST) levels (^Ψ^0.029, ^ΨΨ^0.0006, and ^ΨΨΨ^0.024), **(B)** Alanine aminotransferase (ALT) levels (*0.027, **0.0014, and ***0.0046), **(C)** Circulating levels of free cholesterol (^#^0.0097 and ^##^0.0227), **(D)** Levels of LDL/VLDL are reduced in the DHM group, **(E)** Levels of cholesteryl esters are increased in the DHM group, **(F)** Levels of hepatic triglycerides are increased in the DHM group, **(G)** Levels of circulating triglycerides are reduced in the DHM group (*0.022), and **(H)** immunoprecipitation of CETP expression is reduced with DHM and levels of LCAT are increased in the group fed DHM.

As a measure of overall hepatic health and function following DHM administration, we next analyzed the levels of circulating aspartate and alanine aminotransferases (AST and ALT). As shown in Figure 2A, there was a significant decrease in AST levels in mice receiving oral DHM compared to mice in the EtOH-only group (^Ψ^0.029, ^ΨΨ^0.0006, ^ΨΨΨ^0.024); Figure 2B shows significantly lowered levels of ALT in mice receiving oral DHM when compared to the EtOH-only fed mice (*0.027, **0.0014, and ***0.0046). The levels of circulating lipids were measured and show that DHM administration significantly lowers total free cholesterol levels, (^#^0.0097, ^##^0.0227; Figure 2C). Although not significant, the levels of LDL/VLDL were reduced with DHM administration, like those measured in the No-EtOH group (Figure 2D). When interpreting the results obtained from this study, it is important to note that the LDC diet is considered as a high fat diet, where 35% of calories are derived from FFAs: 23.5 g/L of monounsaturated and 5.2 g/L unsaturated fats. The No-EtOH group is isocaloric to the other groups and therefore is also receiving the high fat diet, which may influence the amount and types of lipids in circulation compared to the EtOH-receiving groups.

Lipid droplets are primarily composed of triglycerides (TGs) and cholesteryl esters (CE) (37). Although TGs are not considered determinants of lipotoxicity (6), conversion of free fatty acids (FFAs) into TGs, as well as FFA utilization in CEs via esterification (40), essentially acts to neutralize the reactivity of and damage caused by excessive FFAs (41, 42). Accordingly, cholesteryl esters and hepatic TG levels were found to be increased in the group receiving DHM (Figures 2E, F), while circulating TG levels were normalized by DHM to levels similar to those measured in the No-EtOH group (*0.022; Figure 2G). Two facilitators of cholesterol exchange and transport are cholesteryl ester transfer protein (CETP) and lecithin-cholesterol acyltransferase (LCAT). CETP is a known mediator in the transfer of cholesteryl esters from HDL to LDL/VLDL (43). LCAT is a key enzyme involved in the esterification of free cholesterol into cholesteryl esters and facilitates the metabolism of cholesterol (44). As demonstrated in Figure 2H, we found that CETP expression was increased in the EtOH-only group compared to the DHM group, and found that LCAT expression was highest in the DHM fed group.

### 3.2. DHM fed mice demonstrate increased colocalization of lipophagy proteins, p62/SQSTM-1, perilipin 1 (PLIN-1) and LC3B

**FIGURE 3:**
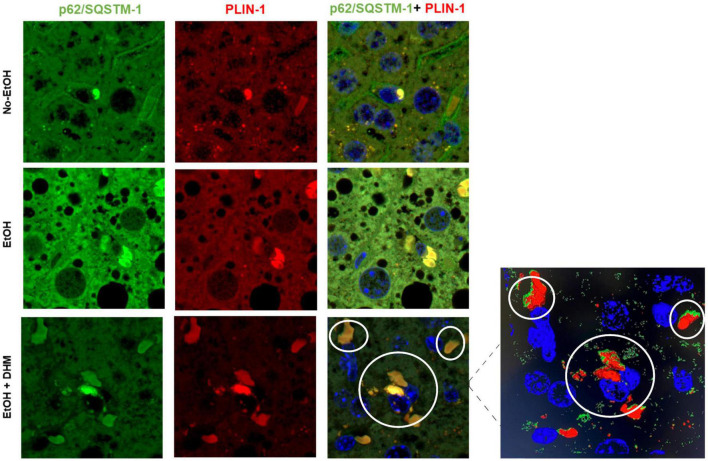
DHM increases ethanol-induced colocalization events between p62/SQSTM-1 and perilipin 1 (PLIN-1). Confocal images (scale bars 5 μm) from the livers of mice receiving the LDC diet confirm the presence and interaction between p62/SQSTM-1 and perilipin 1 (PLIN-1). As shown: p62/SQSTM-1 (green), perilipin 1 (red), and nuclei (blue). Colocalization events are circled between p62/SQSTM-1 + PLIN-1 (yellow/brown). The magnified image from the DHM group highlights the nuclei (blue), p62/SQSTM-1 (green), and PLIN-1 (red) and the magnitude and distribution of colocalization events on lipid droplets.

**FIGURE 4:**
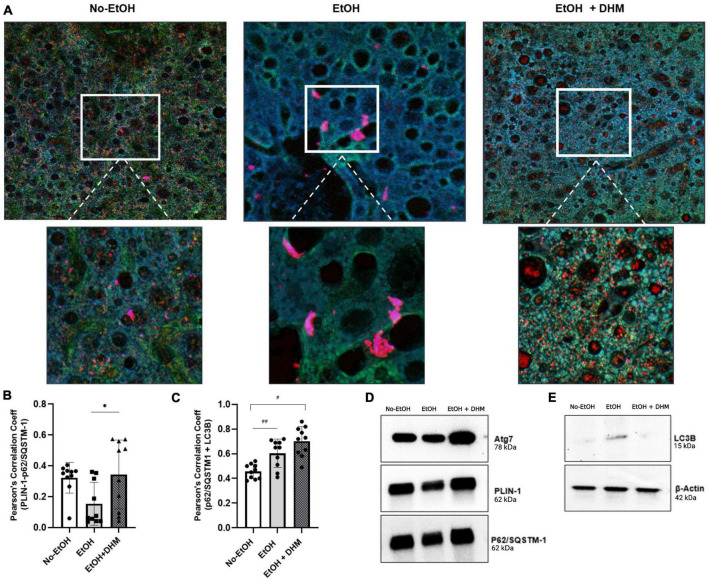
DHM enhances the colocalization and expression of lipophagy-associated proteins in mice exposed to chronic ethanol. DHM administration increases the colocalization and interaction between p62/SQSTM-1 + PLIN-1 + LC3B. **(A)** Confocal images (upper and lower (magnified) scale bars on images are 10 and 5 μm, respectively) show the expression and colocalization of p62/SQSTM-1 (green), PLIN-1 (red), and LC3B (blue) between groups. **(B)** Colocalization of p62/SQSTM-1 + LC3B + PLIN-1 as quantified by Pearson’s Correlation Coefficient (PCC) shows a significant increase in colocalization between PLIN-1 + p62/SQSTM-1 (*0.04; brown puncta). **(C)** Colocalization of p62/SQSTM-1 + LC3B is increased in mice fed EtOH (^##^0.008; cyan puncta) and highest in EtOH + DHM fed mice (^#^< 0.0001). **(D)** Differences in (immunoprecipitated) protein expression levels of Atg7, PLIN-1, and p62/SQSTM-1; **(E)** LC3B protein expression.

Lipid droplet membranes are coated with various components, including lipid droplet-associated proteins belonging to the perilipin family (PLIN-1-5) that assist in the regulation of LD synthesis and cytosolic lipase activity. Lipophagy involves the recruitment of selective autophagy proteins such as sequestosome-1 (p62/SQSTM-1), microtubule-associated protein 1 light chain 3 beta (LC3B), and PLIN-1, which when combined are recognized as defense mechanisms against oxidative stress (45–48). Ethanol is known to trigger the selective interactions of p62/SQSTM-1, LC3B, and PLIN-1 using *in vitro* models during LD clearance (45). To assess the effect of DHM on ethanol-induced interactions between selective autophagy-associated proteins, we analyzed the presence and interactions between p62/SQSTM-1; LC3B; and PLIN-1. We began by confirming the interaction between p62/SQSTM-1 and PLIN-1 using liver sections that were stained for p62/SQSTM-1 (green), PLIN-1 (red), and nuclei (blue). As illustrated in Figure 3 (scale bars 5 μm), we confirmed the presence of and interactions between p62/SQSTM-1 and PLIN-1 in all three groups as shown from their colocalization. Mice receiving ethanol had noticeably higher levels of p62/SQSTM-1 + PLIN-1 interactions than the No-EtOH group (Figure 4B). DHM-fed mice had even greater colocalization events between PLIN-1 and p62/SQSTM-1, where the interactions were widely distributed across LD surfaces, compared to the EtOH-only group (Figure 3 inset).

Autophagy is a diverse mechanism that follows several pathways based on cellular demands. There are over 32 different autophagy-related genes (Atg) that activate the formation of double-membrane structures that deliver cytoplasmic components to lysosomes for degradation. Atg7 is a ligase that has ubiquitin E1-like activity which facilitates interactions and complexations between other autophagy-related genes. These subsequently interact with other Atg proteins, forming a much larger complex that binds to LC3B, a molecule that is essential for autophagosome structure, formation, and cargo recognition (47, 49). LC3B interacts with cargo adaptor protein (p62/SQSTM-1) that binds to poly-ubiquitinated cargo, and is a classical selective autophagy receptor (48).

Selective autophagy occurs when p62/SQSTM-1 and LC3B interact (50), resulting in the formation of an autolysosome that is directed to ubiquitinated PLIN-1 proteins found on LDs, resulting in lipophagy activity. The colocalization of p62/SQSTM-1 + PLIN-1 + LC3B is illustrated in Figure 4A (upper and lower magnified images scale bars are 10 and 5 μm, respectively). Additionally, we quantified levels of interactions by measuring the correlation between two proteins using Pearson’s Correlation Coefficient (PCC) analysis, where values closer to 1.0 confirm the strength of correlation. As shown, interactions between PLIN-1 + p62/SQSTM-1 were significantly higher in mice receiving DHM with a mean PCC of 0.344 (*0.04; brown puncta), compared to the EtOH-only group with a mean PCC of 0.154 (Figure 4B). Our results also show that mice in the group receiving EtOH-only demonstrated an increased colocalization of p62/SQSTM-1 + LC3B (^##^ 0.008; cyan puncta) when compared to the No-EtOH group; the increase was more apparent when comparing the No-EtOH group to mice fed DHM (^#^< 0.0001) (Figure 4C). Our data show that DHM-fed mice had a greater and wider range of colocalization events and activity. Quantification (via immunoprecipitation) of the expression levels of Atg7, PLIN-1, p62/SQSTM-1 were analyzed (Figure 4D), and LC3B: β-Actin (via Western blots) are shown in Figure 4E, supporting the findings from histological colocalization analyses.

### 3.3. DHM reverses ethanol-induced reductions in mitochondrial oxidative phosphorylation activity

**FIGURE 5:**
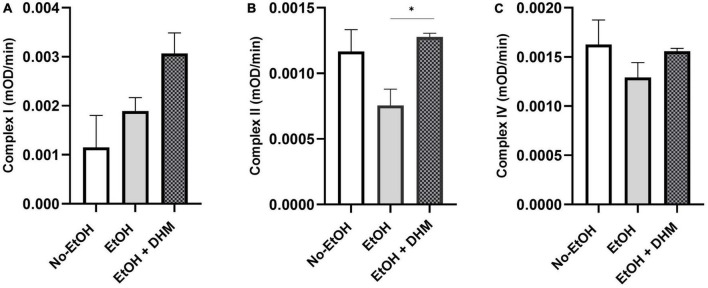
DHM reverses ethanol-induced reductions in mitochondrial function. DHM-fed mice demonstrate improvement in mitochondrial oxidative phosphorylation systems as shown by restored activity in **(A)** complex I, **(B)** complex II (*0.03), and **(C)** complex IV.

Chronic ethanol consumption leads to loss of mitochondrial function and increased production of reactive oxygen species (ROS), promoting oxidative stress, particularly in the mitochondria. Damage, brought on by increases in ROS to mitochondrial proteins and DNA, decreases mitochondrial function due to the breakdown of these complexes (51). Lipid metabolism takes place in the mitochondria, where fatty acids undergo β-oxidation. We measured mitochondrial health by analyzing the activity of complexes I, II, and IV from isolated mitochondria. Our results show that DHM had a significant effect on restoring complex II activity (*0.03; Figure 5B). Although the changes were not significant, DHM led to increases in complex I activity (Figure 5A), while normalizing the activity of complex IV (Figure 5C).

### 3.4. DHM supplementation reduces pro-inflammatory and hematopoietic cytokines

**FIGURE 6:**
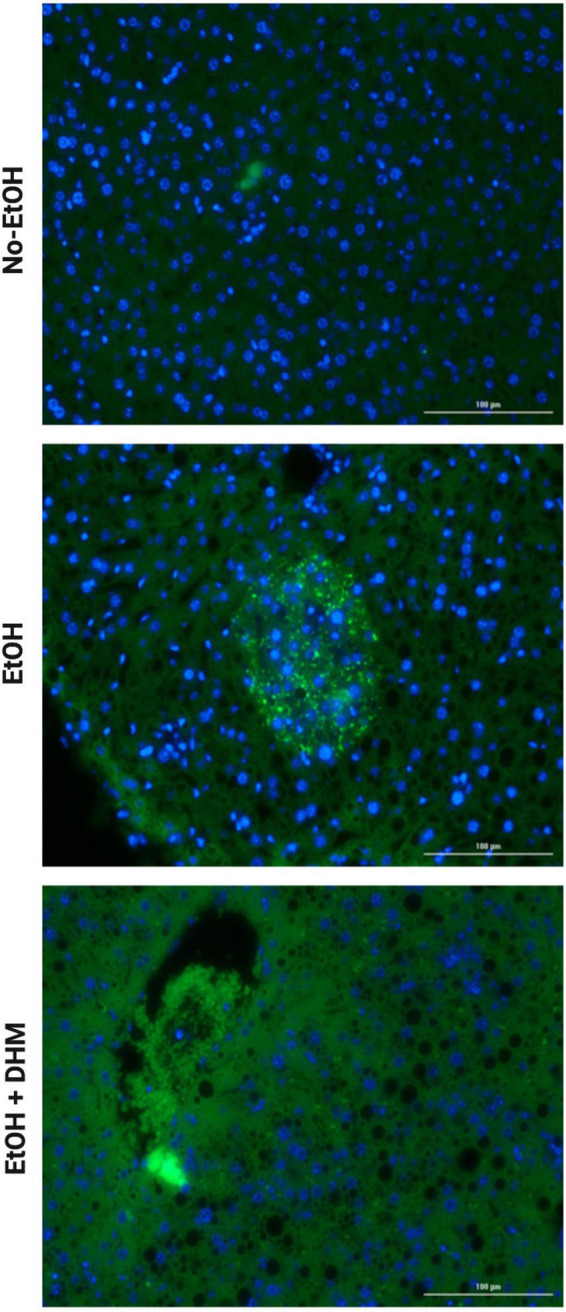
DHM reduces the increase in monocyte infiltration seen in mice exposed to chronic EtOH. Images (scale bars: 100 μm) demonstrating smaller bursts of monocyte infiltration clouds (green puncta) when compared to mice in the EtOH-only group.

**FIGURE 7:**
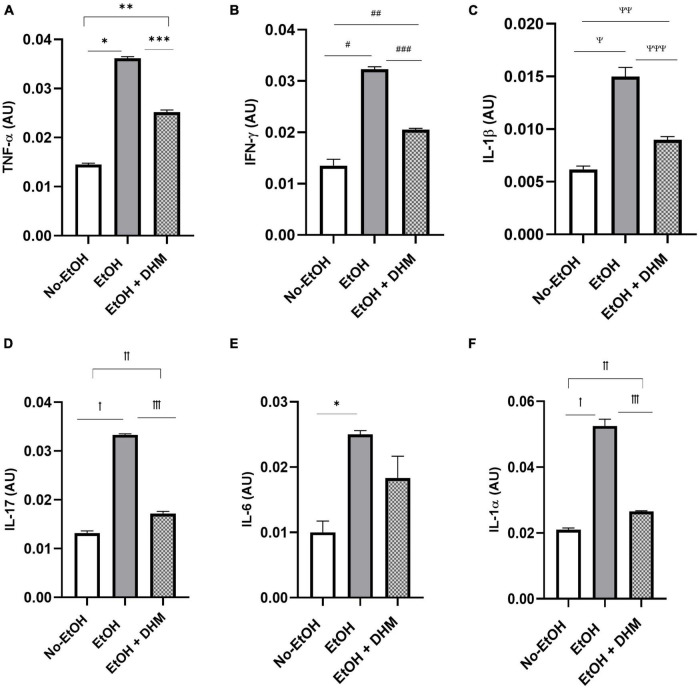
DHM supplementation ameliorates elevations in pro-inflammatory cytokines seen in mice given chronic EtOH. **(A)** DHM-fed mice show significant decreases in levels of TNF-α compared to EtOH-only mice (*, **, and ***< 0.0001). Normalization of levels of **(B)** IFN- γ (^#^< 0.0001, ^##^0.0018, and ^##^0.001) and **(C)** IL-1β (^Ψ^< 0.0001, ^ΨΨ^0.027, and ^ΨΨΨ^0.0007) to those similar to those in the No-EtOH group is shown. DHM-fed mice show significant decreases in **(D)** IL-17 (^ꝉ^< 0.0001, ^ꝉꝉ^0.003, and ^ꝉꝉꝉ^< 0.0001) compared to EtOH-only mice. EtOH-only mice show a significant increase in **(E)** IL-6 (*0.007) expression compared to No-EtOH mice. **(F)** IL-1α (^ꝉ^< 0.0001, ^ꝉꝉ^0.047, and ^ꝉꝉꝉ^< 0.0001) levels are significantly reduced in DHM-fed mice compared to EtOH-only mice.

Next, we investigated the effects of DHM on ethanol-induced inflammation. The livers of mice were stained with CD68, a biomarker for immune cells of the monocyte lineage, such as monocytes and macrophages. As illustrated in Figure 6, mice in the EtOH-only group had larger bursts of monocyte/macrophage infiltration clouds (green puncta) when compared to the No-EtOH and DHM-fed mice. These observations were further confirmed by conducting a cytokine panel assay that measured several different circulating pro-inflammatory cytokines from serum (Supplementary Figure 1). We found that oral DHM administration reduced circulating levels of pro-inflammatory cytokines and immune cell chemokines that are traditionally associated with ethanol-induced inflammation. TNF-α promotes acute inflammation and is one of the critical inflammatory cytokines in ALD progression and liver injury, as it contributes to the production of other pro-inflammatory cytokines (33). DHM supplementation reversed the significant elevations in TNF- α levels in mice receiving ethanol-only as demonstrated in Figure 7A (*, **, and ***< 0.0001), while also normalizing levels of IFN-γ (^#^< 0.0001, ^##^0.0018, and ^##^0.001) closer to those of the No-EtOH group (Figure 7B). DHM receiving mice had reduced levels of IL-1β (^Ψ^< 0.0001, ^ΨΨ^0.027, and ^ΨΨΨ^0.0007) compared to the EtOH-only mice, with levels close to the No-EtOH group (Figure 7C). Although IL-1β is not produced in a healthy liver, it is secreted by activated inflammasomes during excessive alcohol consumption and is an essential cytokine in giving rise to Th17 cells that subsequently secrete IL-17 (52, 53).

**FIGURE 8:**
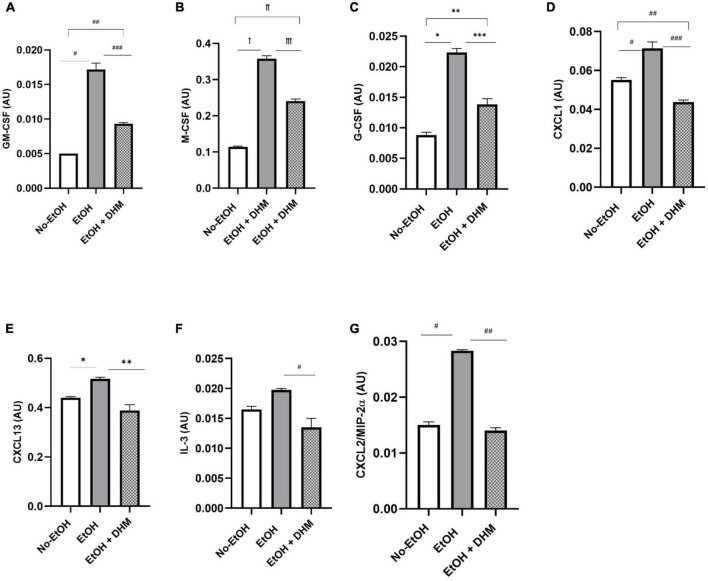
DHM administration reduces levels of hematopoietic cytokines and chemokines which are increased during chronic alcohol consumption. Expression of **(A)** granulocyte-macrophage-colony stimulating factor (GM-CSF; ^#^< 0.0001, ^##^ 0.003, and ^###^0.0001), **(B)** macrophage-colony stimulating factor (M-CSF; ^ꝉ,ꝉꝉ, ꝉꝉꝉ^< 0.0001), and **(C)** granulocyte-colony stimulating factor (G-CSF; *< 0.0001, **0.005, and ***0.0004) significantly decreases with DHM administration. Expression of chemokines **(D)** CXCL1 (^#^< 0.0001, ^##^0.0001, and ^###^< 0.0001) and **(E)** CXCL13 (*0.023 and **0.002) is also reduced in DHM-fed mice. Expression of pro-inflammatory cytokine **(F)** IL-3 (^#^0.035) and pro-inflammatory chemokine **(G)** CXCL2 (^#,##^< 0.0001) is reduced following DHM administration.

Mice receiving EtOH-only had significantly higher levels of circulating IL-17 overall, which was ameliorated with DHM supplementation (Figure 7D; ^ꝉ^< 0.0001, ^ꝉꝉ^0.003, and ^ꝉꝉꝉ^< 0.0001). IL-17 is a potent pro-inflammatory cytokine that has received much attention for its synergistic effects with other inflammation promoting cytokines during ALD pathogenesis that were also reduced in the DHM-fed group, such as IL-6 (Figure 7E; *0.007), IL-1β, and IL-1α (Figure 7F; ^ꝉ^< 0.0001, ^ꝉꝉ^0.047, and ^ꝉꝉꝉ^< 0.0001) (52–54). IL-17 induces the expression of hematopoietic cytokines and chemokines such as granulocyte-macrophage-colony stimulating factor (Figure 8A: GM-CSF; ^#^< 0.0001, ^##^ 0.003, and ^###^0.0001), macrophage-colony stimulating factor (Figure 8B: M-CSF; ^ꝉ,ꝉꝉ, ꝉꝉꝉ^< 0.0001), granulocyte-colony stimulating factor (Figure 8C: G-CSF; *< 0.0001, **0.005, and ***0.0004), neutrophil activating and chemotactic chemokine, CXCL1 (Figure 8D; ^#^< 0.0001, ^##^0.0001, and ^###^< 0.0001), and B-cell recruiting CXCL13 (Figure 8E; *0.023, and **0.002) (53, 55–58). DHM-fed mice had lower levels of IL-3 (Figure 8F; ^#^0.035), a cytokine that amplifies acute inflammation (59) and works in coordination with GM-CSF to promote pathogenic clearance during chronic inflammation (60). Additionally, CXCL2/MIP-2 (macrophage inflammatory protein-2), is synthesized by a variety of immune cells to recruit neutrophils in response to damage and acute liver injury (61). CXCL2 was significantly increased in mice receiving EtOH-only, and that increase was reversed with DHM supplementation (Figure 8G; ^#,##^< 0.0001).

### 3.5. DHM supplementation increased production of protective anti-inflammatory cytokines in mice treated with ethanol

**FIGURE 9:**
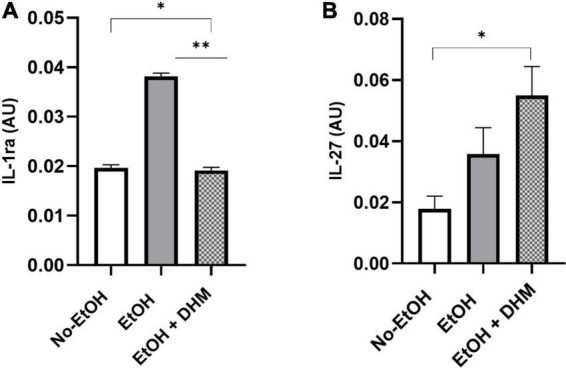
DHM increases production of anti-inflammatory cytokines compared to mice fed EtOH-only. Expression of **(A)** IL-1ra (*, **< 0.0001) is significantly reduced in mice receiving DHM compared to those fed EtOH-only. In addition, levels of circulating **(B)** IL-27 (*0.034) in the DHM-fed group are significantly higher than that in the No-EtOH group.

Mice receiving DHM supplementation had increased anti-inflammatory cytokine levels compared to mice fed EtOH-only. IL-1ra is a receptor antagonist to members of the IL-1 family of pro-inflammatory cytokines, and has neutralizing and protective effects against IL-1 activity (62). Mice in the DHM group had levels of IL-1ra that were nearly identical to the No-EtOH group and significantly lower than the EtOH-only group (Figure 9A; *, **< 0.0001). Following excessive ethanol intake and burn injury, IL-27 has been shown to promote liver regeneration by enhancing liver progenitor cell expansion and differentiation as well as intestinal barrier repair following ethanol intoxication (63, 64). In our study, as shown, DHM-fed mice had significantly higher levels of IL-27 in circulation than that of the No-EtOH group (Figure 9B; *0.034) and nearly twice as much as those in the EtOH-only group.

## 4. Discussion

Despite the detrimental health effects associated with high ethanol intake, individuals continue to partake in excessive drinking behavior, as evidenced by the increasing rates of alcohol sales and alcohol-related mortality and morbidity. ALD is the leading cause of liver disease in the United States, where alcohol accounts for up to 50% of cirrhosis-related mortality (65) and 20% of mortality worldwide (66). Therefore, targeting ethanol-induced steatosis and the mechanisms that lead to the dysregulation of lipid homeostasis are key for preventing lipotoxicity and the systemic metabolic dysfunction that eventually affects multiple organ systems (6). Considering the growing interest and consumer preference for herbal therapies (i.e., polyphenols and flavonoids), the present study tested the hypothesis that the bioactive polyphenolic-flavonoid DHM, improves ethanol-induced lipid imbalance and steatosis in part by restoring lipophagy activity and reducing pro-inflammatory cytokines. The data presented here supports the hypothesis that DHM can counteract the progression of ALD pathology caused by damage due to inflammation and the dysregulation in lipid homeostasis due to chronic ethanol consumption.

The liver is the primary site for the breakdown of ethanol and is one of the major organs for lipid metabolism and is, therefore, highly susceptible to damage and lipotoxicity. Elevated levels of lipids is a major factor that leads to hepatic injury caused by lipotoxicity and oxidative stress. Cellular defense mechanisms neutralize FFAs via their conversion into TGs through esterification. Lipid droplets are primarily composed of TGs and CEs, acting as energy stores, subsequently minimizing the lipotoxicity of FFAs that would otherwise occur in the cell (37, 42). Cholesteryl esters are reverse transported to the liver from circulation and peripheral tissues via high-density lipoproteins, where they are stored in LDs or metabolized for bile acid synthesis (67). The increased levels of CEs, hepatic TGs, and LD size in our study, combined with the reduction in circulating TGs, suggest increased synthesis and hepatic sequestration of TGs and CEs in the DHM-fed mice. Taken together, the results from our study indicate the possibility of increased FFA neutralization and containment in LDs as a protective measure against lipotoxicity by FFAs. Chronic ethanol consumption disturbs metabolic flux through various pathways. As mentioned earlier, the LDC diet is regarded as a high fat diet, providing excess dietary free fatty acids to all groups. Future studies will also consider the effect of a high fat diet when measuring circulating lipid content in the No-EtOH group(s) and comparing them to EtOH-fed group(s).

Chronic ethanol consumption alters metabolic processes, including hepatocyte LD properties that include LD membrane protein composition, resulting in increased size and differential tissue distribution (38). Lipophagy, a subtype of macroautophagy, is associated with the degradation of LDs via engulfment by autophagosomes and subsequent fusion with lysosomes. Ethanol can stimulate autophagy through multiple mechanisms, including the modulation of mammalian target of rapamycin (mTOR) through AMPK signaling pathways. Excessive ethanol consumption is associated with decreased AMPK activation, which in turn activates mTOR in the liver and inhibits autophagy (21, 68). Previous work found that DHM can improve autophagy activity by activating AMPK, inhibiting mTOR, and reversing ethanol-induced AMPK-deficiency (21, 69, 70). Results from the current study demonstrate the downstream activity of AMPK-autophagy related activity and offer a glimpse into the possible downstream effects on lipophagy, as demonstrated by the enhanced expression and interactions between lipophagy protein complexes p62, LC3B, and PLIN-1.

Members of the PLIN family of LD-associated proteins are essential for regulating triglyceride synthesis, packaging TGs into LDs, and lipolysis. PLIN-1 positively contributes to the formation of larger LDs and is expressed on the membranes of larger, more mature LDs (36, 38, 71, 72). Studies have also shown that lipolysis is enhanced and regulated via proteasomal degradation of PLIN1 (73–75). Activation of protein kinase A (PKA) via AMPK signaling leads to the phosphorylation of PLINs (76), which are then subjected to ubiquitination and are tagged for proteasomal degradation. This results in effective priming of LD surfaces for recognition and recruitment of autolysosomal bodies through the activities of selective mechanisms such as those directed by p62/SQSTM-1 (46, 77). Lipolysis and lipophagy are tandem pathways in hepatocytes. Lipolysis is the process in which FFAs are released from TGs, which takes place during lipophagy (in lysosomes), and preferentially targets the degradation of large LDs. The increased presence of PLIN-1 and interactions with p62 and LC3B in the DHM-fed mice demonstrates the possibility of enhanced lipophagic activity, which potentially results in greater lipid clearance over time.

Released FFAs are then further broken down in the mitochondria, where they undergo β-oxidation. This system works in tandem with the oxidative phosphorylation system (OXPHOS), which is located in the mitochondrial inner membrane, composed of four respiratory chain complexes (I-IV), and is key for driving ATP production (51). As such, mitochondrial health and function is determined by the analysis of OXPHOS-complex activity (78). Previous studies have demonstrated the effect of DHM on mitochondrial health, effectively reversing stress-induced deficiencies in mitochondrial function (19, 21, 79, 80). Our data demonstrates a positive effect of DHM on mitochondrial function restoration, particularly in Complex II. The increase in lipophagy activity is possible when mitochondrial function is efficient, as measured by complexes I, II, and IV. This data further supports the potential benefit of DHM on reversing ethanol-induced OXPHOS deficiencies and in turn, improving overall function.

In addition to direct induction of oxidative stress-induced inflammation, alcohol disrupts gut permeability, causing endotoxin/lipopolysaccharide (LPS) translocation to interact with TLR4 which results in the generation of inflammatory cytokines via NF-κB signaling pathway activation (31, 81). The increased oxidative stress and increased TLR4/NF-κB transcription upregulates and activates inflammasome, an intracellular protein complex that leads to the cleavage of pro-inflammatory cytokines like IL-1β (82). In ALD, pro-inflammatory cytokines such as TNF-α, IL-1β, and IL-17 are produced by alcohol-induced activation of liver innate immunity (30). In addition, leukocyte chemoattractants and hematopoietic cytokines can recruit and proliferate immune cells in the liver, exacerbating the inflammatory response. IL-3 is a hematopoietic cytokine that regulates the differentiation, proliferation, and survival of various immune cells. DHM has been shown to suppress the production of hematopoietic cytokines that regulate the differentiation, proliferation, and survival of various immune cells such as IL-3, M-CSF, and G-CSF in LPS-stimulated macrophages (83). Furthermore, our study shows that DHM decreases the expression of CXCL1 and CXCL13, chemokines that are involved in the recruitment and activation of neutrophils and B cells, respectively, in hepatic inflammatory processes (84, 85). Evidence shows that inflammation plays an essential role in the initiation and progression of ALD (86). Results from our study support the published reports on the immuno-modulatory activity of DHM, and offer a glimpse on the protective effects of DHM against the damaging effects of ethanol-induced inflammation and injury (87, 88). The mechanism of action of DHM in reducing inflammation in ALD is believed to be multifactorial. As demonstrated in this study, DHM supplementation led to significant decreases in inflammatory signaling through reductions in the prominent pro-inflammatory cytokines associated with ALD pathology. Interestingly, DHM supplementation led to a significant increase in IL-27, a cytokine that has demonstrated protective action on the gut barrier by promoting anti-inflammatory functions, regenerative activity in the liver and intestines, and promoting intestinal barrier repair following ethanol intoxication and burn injury (63, 64).

The results from our study align with various animal and human studies investigating DHM for its robust antioxidant activity (79, 89), ability to reverse dyslipidemia (18, 21, 90, 91), having anti-alcohol intoxication effects (92), and amelioration of non-alcohol-associated fatty liver disease (19).

With the rates of ethanol-related health effects continuing to rise, particularly ALD, the need for therapeutic intervention is imperative. DHM is a natural compound that is widely available as a dietary supplement and has demonstrated the potential to mitigate the progression of ALD development caused by disruptions in lipid metabolism and transport in mice. As a natural product that is readily and commercially available, our findings help set the stage for the rapid advancement of DHM to improve liver health against the damaging effects of excessive ethanol consumption.

## Data availability statement

The raw data supporting the conclusions of this article will be made available by the authors, without undue reservation.

## Ethics statement

The animal study was reviewed and approved by the Institutional Animal Care and Use Committee (IACUC).

## Author contributions

IJ-U served as the project lead and conceptualized the contents and the project related to this manuscript. EC and DD contributed to the conception and design of the study. AI, MZ, MV, NS, SS, SC, ZZ, JW, and LA contributed either by gathering, analyzing, and/or interpreting data, as well as to the writing and intellectual content of the manuscript. All authors read and approved the final manuscript.

## Supplementary material

Supplementary Figure 1 Cytokine Profile Arrays. Cytokines were measured from serum using a cytokine profile array such as the one shown here, where each dot blot represents treatment groups (U0781: No-EtOH; U0782: EtOH; U0784: EtOH + DHM).
