# Supplementary figures and images for: Dihydromyricetin supplementation improves ethanol-induced lipid accumulation and inflammation
Source: Front Nutr. 2023 Aug 23;10:1201007. doi: 10.3389/fnut.2023.1201007 (PMC10481966; doi:10.3389/fnut.2023.1201007)

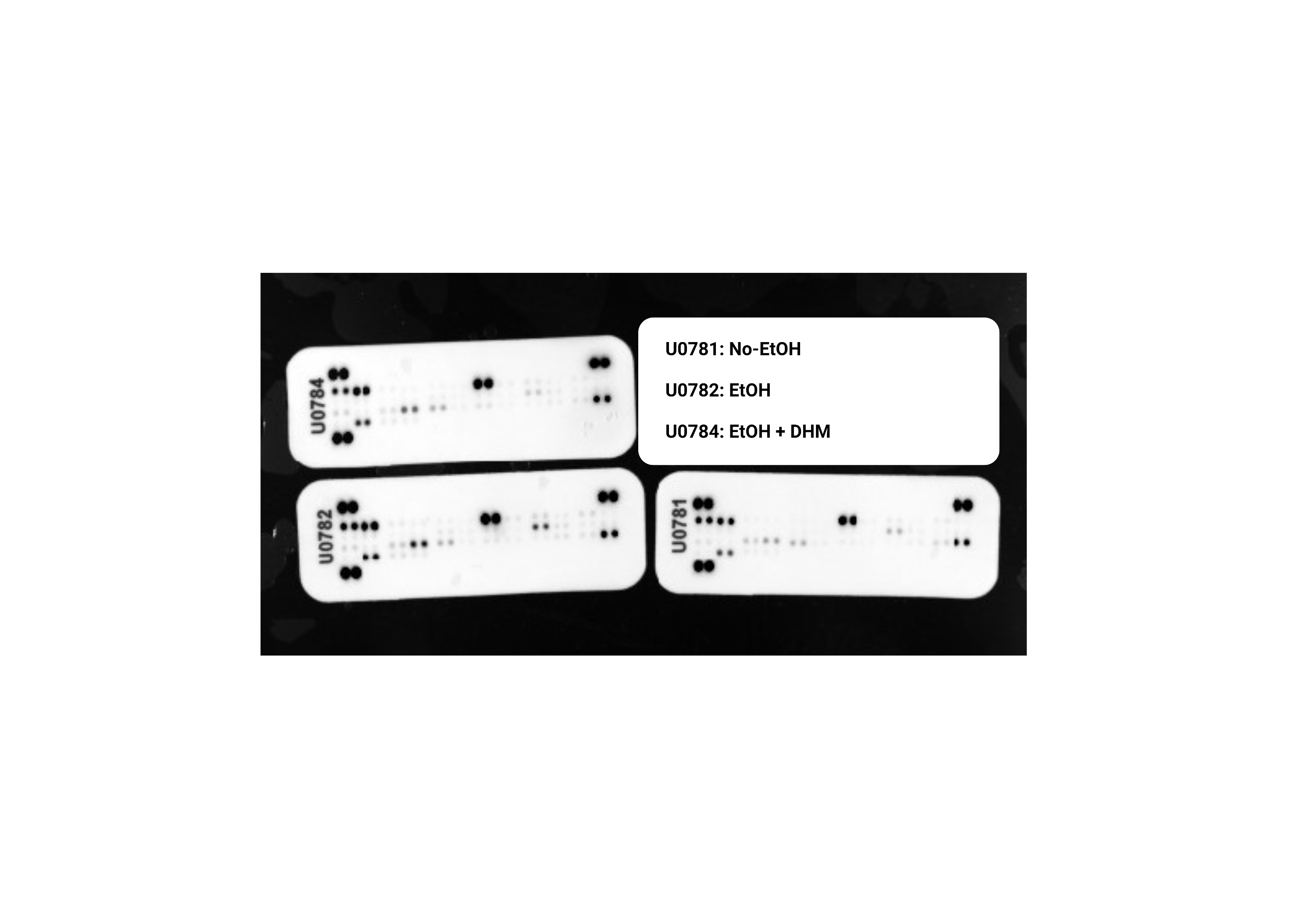

Supplement: Supplementary Figure 1 — Cytokine Profile Arrays. Cytokines were measured from serum using a cytokine profile array such as the one shown here, where each dot blot represents treatment groups (U0781: No-EtOH; U0782: EtOH; U0784: EtOH + DHM). [file Image_1.JPEG]
